# Continental Treatment of Neuralgia

**Published:** 1843-11

**Authors:** 


*Continental Treatment of Neuralgia.*—Dr. Schleiser, of Peitz, has prescribed, with success, to patients with abdominal neuralgia, but whose circumstances would not permit of their visiting any watering-place, the use of an artificial mineral water, resembling that of Eger, in Bohemia, and made as follows:—*R. Filtered spring-water*, a pint; *diluted sulphuric acid*, two drachms and a half; *hydrochloric acid*, twenty drops. Mix, and add *bicarbonate of soda*, forty-five grains. The bottles are then to be sealed up without delay, and kept cool; one or two pints may be drunk daily. In hepatic neuralgia, Dr. Schleiser depends much on the effects of belladonna; in cases where great irritability of the stomach is present, he finds nitrate of silver suitable, combined with morphia.

*Rust's Magazin.*

Morphia has been an ordinary remedy for neuralgia, the cure of which it may, in certain cases, effect; but a French practitioner, Rougier, has advised the adoption of an ingenious method, which he says will prove the completeness and permanance of the cure. After the apparent removal of the disease by the morphia, he administers successive small doses of strychnia, gradually increasing the amount of the doses and abridging the intervals between them. Now, if the cure have been complete, the tremors and other characteristic effects of the strychnia go on diminishing in intensity from the first, notwithstanding the increasing strength and frequency of the doses; but if otherwise a contrary result happens, and the effects of the strychnia increase in intensity.—*London Lancet*, August, 1843, from *L'Experience.*

